# Risk Factors for Dual Burden of Severe Maternal Morbidity and Preterm Birth by Insurance Type in California

**DOI:** 10.1007/s10995-021-03313-1

**Published:** 2022-01-18

**Authors:** Alison M. El Ayadi, Rebecca J. Baer, Caryl Gay, Henry C. Lee, Juno Obedin-Maliver, Laura Jelliffe-Pawlowski, Audrey Lyndon

**Affiliations:** 1 Department of Obstetrics, Gynecology and Reproductive Sciences, University of California, San Francisco, 550 16th Street, 3rd Floor, San Francisco, CA 94158 USA; 2 Department of Pediatrics, University of California, San Diego, San Diego, CA USA; 3 Department of Family Health Care Nursing, University of California, San Francisco, San Francisco, CA USA; 4 California Preterm Birth Initiative, University of California, San Francisco, San Francisco, CA USA; 5 Division of Neonatal & Developmental Medicine, Department of Pediatrics, Stanford University, Stanford, CA USA; 6 California Perinatal Quality Care Collaborative, Stanford, CA USA; 7 Department of Obstetrics and Gynecology, Stanford University, Stanford, CA USA; 8 Department of Epidemiology and Biostatistics, University of California, San Francisco, San Francisco, CA USA; 9 Rory Meyers College of Nursing, New York University, New York, NY USA

**Keywords:** Severe maternal morbidity, Preterm birth, Health disparities, Insurance coverage

## Abstract

**Objectives:**

Among childbearing women, insurance coverage determines degree of access to preventive and emergency care for maternal and infant health. Maternal-infant dyads with dual burden of severe maternal morbidity and preterm birth experience high physical and psychological morbidity, and the risk of dual burden varies by insurance type. We examined whether sociodemographic and perinatal risk factors of dual burden differed by insurance type.

**Methods:**

We estimated relative risks of dual burden by maternal sociodemographic and perinatal characteristics in the 2007–2012 California birth cohort dataset stratified by insurance type and compared effects across insurance types using Wald Z-statistics.

**Results:**

Dual burden ranged from 0.36% of privately insured births to 0.41% of uninsured births. Obstetric comorbidities, multiple gestation, parity, and birth mode conferred the largest risks across all insurance types, but effect magnitude differed. The adjusted relative risk of dual burden associated with preeclampsia superimposed on preexisting hypertension ranged from 9.1 (95% CI 7.6–10.9) for privately insured to 15.9 (95% CI 9.1–27.6) among uninsured. The adjusted relative risk of dual burden associated with cesarean birth ranged from 3.1 (95% CI 2.7–3.5) for women with Medi-Cal to 5.4 (95% CI 3.5–8.2) for women with other insurance among primiparas, and 7.0 (95% CI 6.0–8.3) to 19.4 (95% CI 10.3–36.3), respectively, among multiparas.

**Conclusions:**

Risk factors of dual burden differed by insurance type across sociodemographic and perinatal factors, suggesting that care quality may differ by insurance type. Attention to peripartum care access and care quality provided by insurance type is needed to improve maternal and neonatal health.

**Supplementary Information:**

The online version contains supplementary material available at 10.1007/s10995-021-03313-1.

## Significance

*What is already known on this topic?* Risk for dual burden of severe maternal morbidity and preterm birth differs by insurance type. Insurance influences U.S. maternal and infant health care access and quality and may contribute to the relative importance of factors associated with dual burden.

*What this study adds* The differences identified in dual burden risk by insurance type may increase certain women’s risks of dual burden of severe maternal morbidity and preterm birth. Our findings point to the need to review care quality by insurance type and ensure high quality peripartum care regardless of insurance type to improve maternal and neonatal health.

## Introduction

Insurance coverage is an important contributor to U.S. health disparities, with substantial differences in health status and outcomes observed not only among individuals without insurance coverage compared to individuals with coverage but also across different categories of insurance (Dickman et al., 2017; Freeman et al., 2008; Griffith et al., 2017; Hadley, 2007; McWilliams 2009). Among the insured, coverage influences health status, outcomes, and survival through determining access to health education, clinical and social preventive services, and clinical services for chronic and acute conditions (Chikani et al., 2015; Sommers et al., 2017; Woolhandler & Himmelstein, 2017). Uninsured non-elderly American adults are sicker, less likely to receive preventive services, and more likely to receive lower quality medical care than insured individuals when hospitalized for chronic and acute conditions (Institute of Medicine (US) Committee on the Consequences of Uninsurance, 2002; Woolhandler & Himmelstein, 2017). Even within the same facility, insurance type has been identified as an important factor determining quality of care (Spencer et al., 2013). Health insurance is a particularly critical factor for childbearing women, as it may limit preventive and emergency care for conditions that contribute to adverse maternal or infant outcomes (Johnson et al., 2006). Given the high costs and consequences of inadequate care access and low quality care during this vulnerable time, ensuring high quality coverage is of particular importance (Johnson et al., 2006; Sakala & Corry, 2008).

Insurance type is an important independent risk factor for two significant causes of poor maternal and neonatal health: severe maternal morbidity and preterm birth. Severe maternal morbidity (SMM) occurs in approximately 2.4% of U.S. births (Callaghan et al., 2012; Carmichael et al., 2021; Geller et al., 2018; Grobman et al., 2014; Lyndon et al., 2019). SMM has increased nearly 200% over recent decades, from 49.5 per 10,000 childbirth hospitalizations in the early 1990s to 146.6 in 2015 (Centers for Disease Control & Prevention, 2018; Fingar et al., 2018). SMM has profound physical, psychological, social, and financial consequences for women and their families.(Norhayati et al., 2015) Compared to women with private insurance, Californian women with Medi-cal or other insurance (Indian Health Service, CHAMPUS or TRICARE, other local, state, or federal insurance, or charity), or no insurance, have a 24–29% increased risk of SMM (Lyndon et al., 2012). Several studies have identified a significant relationship between SMM and preterm birth, with over one-quarter of women with SMM delivering preterm in national and state-level studies (Kilpatrick et al., 2016; Lyndon et al., 2019; Reddy et al., 2015).

Preterm birth (< 37 weeks gestational age) is the principal contributor to neonatal and child mortality, occurring in approximately one in ten births globally, including in the United States (Harrison & Goldenberg, 2016; Martin et al., 2017). Infants born preterm are at increased risk of respiratory, cardiovascular, neurologic, and gastrointestinal morbidities, and long-term respiratory, sensory, emotional, and neurocognitive challenges (Frey & Klebanoff, 2016; Manuck et al., 2016). In the National Inpatient Sample, the largest all-payor inpatient care database in the United States, Medicaid was the payor for over half of preterm births, and Medicaid-covered preterm births had double the neonatal rehospitalization of preterm births covered by commercial insurers (Barradas et al., 2016).

Women and infant dyads with dual burden of SMM and preterm birth are a uniquely vulnerable group, experiencing high physical morbidity combined with psychological and psychosocial concerns for families, such as increased risk of postpartum depression or post-traumatic stress disorder, and sequelae of these conditions (Elmir & Schmied, 2016; Elmir et al., 2012; Fenech & Thomson, 2014; Lefkovics et al., 2014). Prior research has highlighted differences in risk of the dual burden of SMM and preterm birth that exist by insurance type. Compared to women with private insurance, risk of dual burden was 20% higher for women with Medi-Cal, California’s Medicaid program, or other insurances, and 30% higher for uninsured women (Lyndon et al., 2019). Given the differences in maternal and infant health care access and quality by insurance type, we sought to understand how the associations between sociodemographic and perinatal factors and risk of dual burden of SMM and preterm birth differed by insurance type using a large in-patient administrative dataset from California.

## Materials and Methods

**Fig. 1:**
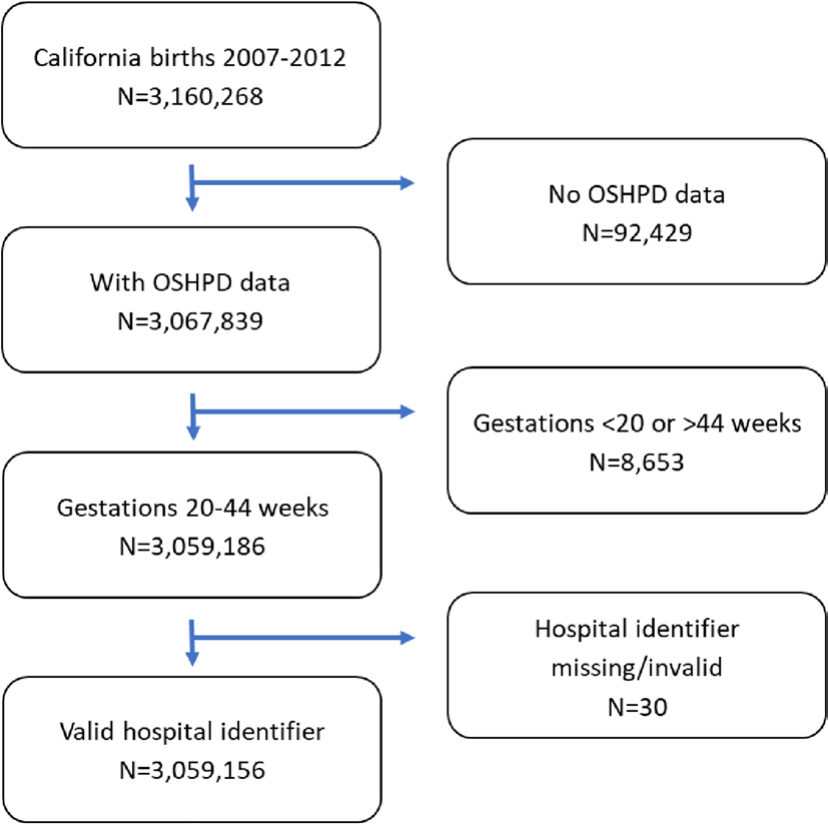
Flowchart describing analytic sample. *Notes* OSHPD: California Office of Statewide Health Planning and Development

We conducted a retrospective analysis of 3,059,156 California live births occurring between January 1, 2007, and December 31, 2012. Eligible births in the California Office of Statewide Health Planning and Development birth cohort database were births with gestation lengths between 20 and 44 weeks and a valid hospital identifier (Fig. 1). This birth cohort database contains data from linked birth and infant death certificates, detailed information on maternal and infant characteristics, and hospital discharge diagnoses and procedures recorded within one year prior to and nine months following birth. Diagnosis and procedure codes were based on the International Classification of Diseases, 9th Revision, Clinical Modification (ICD-9) (American Medical Association, 2008).

Our composite outcome was dual burden of SMM in combination with preterm birth. We defined preterm birth as having occurred at less than 37 weeks gestation per best obstetric estimation recorded on the birth certificate. We categorized inpatient diagnosis and procedural codes associated with unexpected maternal outcomes of labor and birth as SMM using the published Centers for Disease Control and Prevention (CDC) algorithm(Centers for Disease Control & Prevention. Severe Maternal Morbidity Indicators & Corresponding ICD-9-CM Codes during Delivery Hospitalizations, 2016) (Supplemental Table 1). For comprehensive assessment, we followed the CDC definition and included SMM that occurred during childbirth hospitalization and SMM identified on postpartum readmission within 45 days of birth (Carmichael et al., 2021). We excluded morbidity cases that were not severe through restricting SMM during childbirth hospitalization to women with length of stay ≥ 90th percentile operationalized separately for vaginal birth (three days), primary cesarean (five days), and repeat cesarean (four days) (Callaghan et al., 2012). No length of stay restriction was applied to SMM on postpartum readmission.

Analyses were stratified by insurance type at childbirth (Medi-Cal, private, uninsured, and other^1^) to explore the relationships between maternal sociodemographic and perinatal characteristics and outcome of dual burden of SMM and preterm birth within each insurance group. We first estimated the rate of dual burden per 10,000 births overall and by maternal sociodemographic and perinatal characteristics. We then calculated risks of dual burden by maternal factors using modified Poisson regression models for binary outcomes with a robust error variance for hospital to obtain relative risks and 95% confidence intervals (CI). Predictors included sociodemographic and perinatal characteristics selected a priori from our conceptual model of predictors and consequences of preterm birth and severe maternal morbidity and predictors available within the administrative dataset (Table 1) (Lyndon et al., 2019).

**Table 1:** Sociodemographic and perinatal covariates included in stratified analyses of dual burden of severe maternal morbidity and preterm birth, California 2007–2012

Category	Measure	Source
**Sociodemographic characteristics**		
	Age	BC
	Race/ethnicity *(white non-Hispanic, Black non-Hispanic, Hispanic, Asian non-Hispanic, and other non-Hispanic)*	BC
	Educational attainment *(*< *12 years, 12 years,* > *12 years)*	BC
	Level of country urbanity/rurality^a^	BC
**Perinatal factors**		
Prenatal factors	Pre-pregnancy body mass index (BMI), calculated from pre-pregnancy weight and height	BC
	Adequacy of prenatal care *(inadequate: received* < *50% of expected visits, intermediate: received 50–79% of expected visits, adequate/adequate plus: received 80% of expected visits or more)*^*b*^	BC
	Smoking status *(ICD-9 649.0)*	BC & PDD
Pregnancy factors	Mode of birth *(vaginal vs. cesarean)*	BC
	Gestation *(singleton or multiple gestation)*	BC
Obstetric comorbidities	Preexisting hypertension without progression to preeclampsia *(ICD-9 s 642.0 Benign essential hypertension; 642.1 Hypertension secondary to renal disease; 642.2 Other preexisting hypertension)*	PDD
	Preexisting hypertension with progression to preeclampsia *(ICD-9 642.7 Preeclampsia or eclampsia superimposed on preexisting hypertension)*	PDD
	Gestational hypertension without progression to preeclampsia *(ICD-9 642.3 Transient hypertension of pregnancy)*	PDD
	Gestational hypertension with progression to preeclampsia *(ICD-9 642.4 Mild or unspecified preeclampsia, 642.5 Severe preeclampsia)*	PDD
	Preexisting diabetes *(ICD-9 648.0 Diabetes mellitus; 250 Diabetes mellitus)*	PDD
	Gestational diabetes *(ICD-9 648.8 Abnormal glucose tolerance complicating pregnancy)*	PDD

*BC* birth certificate, *PDD* patient discharge data

^a^Federal Information Processing Standard (FIPS) county codes (1–6)

^b^Kotelchuck Adequacy of Prenatal Care Utilization Index (Harrison & Goldenberg, 2016); ICD-9: International Classification of Diseases, 9th Revision, Clinical Modification

Sociodemographic and pregnancy-related characteristics were described within each payor type by dual morbidity burden (rate per 10,000). We estimated the unadjusted relative risks of dual burden of SMM and preterm birth for each predictor of interest, stratified by insurance type, and adjusted relative risks in stratified models. We conducted pairwise comparisons between insurance type using Wald Z-statistics.

All analyses were performed using Statistical Analysis Software version 9.4 (Cary, NC), and differences where p < 0.05 were considered statistically significant. The study was approved by the Committee for the Protection of Human Subjects within the Health and Human Services Agency of the State of California. Data used for the study were obtained by [Institute] at [University] in June 2016. We employ the terms woman and maternal referring to biological sex and related to pregnancy, while acknowledging that not all pregnant/birthing individuals will identify with these terms (Moseson et al., 2020).

## Results

Our final analytic sample included 3,059,156 women, of which there were 45,427 cases of severe maternal morbidity (1.5%) and 267,585 cases of preterm birth (8.7%), with 11,417 cases representing dual burden of SMM and preterm birth (0.37%). Sociodemographic characteristics and perinatal factors of the overall sample are presented by insurance type in Table 2. Dual burden of SMM and preterm birth ranged from 0.36% of privately insured births and 0.38% of Medi-Cal-insured births to 0.41% among uninsured births and births covered by other insurance, respectively (Table 3). Distributions of births and preterm birth indications by gestational age category are presented for each insurance type in Table 4.

**Table 2:** Sociodemographic and perinatal characteristics of births by insurance type, California, 2007–2012

Characteristic	Medical		Private		Uninsured		Other^a^	
	(n = 1,462,463)		(n = 1,431,956)		(n = 64,507)		(n = 100,149)	
	n	%	n	%	n	%	n	%
**Sociodemographic characteristics**								
Maternal age								
< 18	65,973	4.5	15,583	1.1	2336	3.6	2865	2.9
18–25	671,424	45.9	253,134	17.7	18,257	28.3	36,148	36.1
26–35	560,762	38.3	799,134	55.8	31,568	48.9	45,873	45.8
> 35	164,250	11.2	364,015	25.4	12,336	19.1	15,252	15.2
Race/ethnicity								
White, non-Hispanic	190,618	13.0	579,269	40.5	8607	13.3	30,690	30.6
Hispanic	985,156	67.4	420,775	29.4	32,857	50.9	39,322	39.3
Black, non-Hispanic	93,543	6.4	58,687	4.1	2658	4.1	10,792	10.8
Asian, non-Hispanic	82,926	5.7	266,518	18.6	17,330	26.9	11,852	11.8
Other, non-Hispanic	110,220	7.5	106,707	7.5	3055	4.7	7493	7.5
Education								
< 12 years	609,873	43.4	83,068	6	14,419	23.8	15,943	16.4
12 years	468,283	33.3	264,557	19.2	13,705	22.6	30,490	31.4
> 12 years	328,700	23.4	1,032,843	74.8	32,568	53.7	50,562	52.1
Urban/rural status^b^								
1 (most urban)	874,356	59.9	934,009	65.5	42,787	73.9	66,519	66.9
2	150,251	10.3	207,198	14.5	4875	8.4	10,113	10.2
3	333,390	22.9	220,665	15.5	6082	10.5	19,399	19.5
4	71,031	4.9	46,845	3.3	3465	6.0	2646	2.7
5	21,467	1.5	12,942	0.9	512	0.9	557	0.6
6 (most rural)	8311	0.6	5356	0.4	186	0.3	221	0.2
**Perinatal factors**								
*Prenatal factors*								
Maternal pre-pregnancy BMI								
< 18.5	74,820	5.5	64,321	4.8	4574	8.5	4661	4.9
18.5–24.9	577,474	42.8	741,382	54.8	29,650	55.1	47,716	50.1
25.0–29.9	375,164	27.8	314,887	23.3	12,156	22.6	23,782	25.0
≥ 30	322,330	23.9	232,738	17.2	7459	13.9	19,136	20.1
Prenatal care								
Inadequate	368,351	25.8	159,200	11.3	19,789	32.1	17,342	17.7
Intermediate	130,321	9.1	158,831	11.3	6362	10.3	8147	8.3
Adequate/adequate plus	930,921	65.1	1,090,559	77.4	35,404	57.5	72,441	74
Smoked during pregnancy								
Yes	97,167	6.6	32,985	2.3	3223	5.0	5282	8.2
No	1,365,296	93.4	1,398,971	97.7	61,284	95.0	94,867	147.1
* Pregnancy factors*								
Parity and mode of birth								
Primiparous vaginal	364,947	25.0	410,145	28.7	20,997	31.5	21,794	23.0
Multiparous vaginal	621,452	42.5	536,124	37.5	24,604	37	38,987	41.2
Primiparous primary cesarean	151,392	10.4	206,884	14.5	8289	12.5	12,210	12.9
Multiparous primary cesarean	90,416	6.2	84,814	5.9	3444	5.2	6356	6.7
Repeat cesarean	233,835	16	192,926	13.5	9220	13.9	15,221	16.1
Pregnancy type								
Singleton	1,430,425	97.8	1,373,532	95.9	62,789	97.3	97,034	96.9
Multiple gestation	32,038	2.2	58,424	4.1	1718	2.7	3115	3.1
*Obstetric co-morbidities*								
Hypertension								
No HTN	1,354,596	99	1,319,204	98.6	61,002	99.5	92,494	98.7
Preexisting HTN without preeclampsia	13,440	1	18,356	1.4	333	0.5	1216	1.3
Preexisting HTN with preeclampsia	6992	0.5	7506	0.6	195	0.3	559	0.6
Gestational HTN without preeclampsia	31,293	2.3	32,960	2.5	1096	1.8	2403	2.6
Gestational HTN with preeclampsia	47,448	3.5	46,050	3.4	1607	2.6	2729	2.9
Diabetes								
No diabetes	1,329,739	90.9	1,288,453	90	60,881	94.4	92,519	92.4
Preexisting diabetes	13,801	0.9	10,378	0.7	260	0.4	831	1.3
Gestational diabetes	118,923	8.1	133,125	9.3	3366	5.2	6799	10.5

*BMI* body mass index, *HTN* hypertension

^a^Other insurance includes: Indian Health Service, CHAMPUS or TRICARE, other local, state, or federal insurance, or charity

^b^Federal Information Processing Standard categories

**Table 3:** Dual burden of severe maternal morbidity and preterm birth by sociodemographic and perinatal characteristics and insurance type, California, 2007–2012

Characteristic	Medi-cal		Private		Uninsured		Other insurance^a^	
	n = 1,462,463		n = 1,431,956		n = 64,507		n = 100,149	
	n	%	n	%	n	%	n	%
Overall	5541	0.38	5204	0.36	266	0.41	406	0.41
**Sociodemographic characteristics**								
Maternal age								
< 18	177	0.27	56	0.36	12	0.51	11	0.38
18–25	1920	0.29	613	0.24	86	0.47	94	0.26
26–35	2311	0.41	2429	0.30	95	0.30	187	0.41
> 35	1130	0.69	2100	0.58	73	0.59	113	0.74
Race/ethnicity								
White, non-Hispanic	714	0.37	1889	0.33	58	0.67	112	0.36
Hispanic	3231	0.33	1458	0.35	124	0.38	141	0.36
Black, non-Hispanic	814	0.87	412	0.70	33	1.24	95	0.88
Asian, non-Hispanic	261	0.31	936	0.35	28	0.16	29	0.24
Other, non-Hispanic	521	0.47	509	0.48	32	1.05	29	0.39
Education								
< 12 years	2201	0.36	338	0.41	87	0.60	67	0.42
12 years	1772	0.38	927	0.35	77	0.56	121	0.40
> 12 years	1267	0.39	3655	0.35	89	0.27	193	0.38
Urban/rural status^b^								
1 (most urban)	3316	0.38	3368	0.36	141	0.33	275	0.41
2	620	0.41	835	0.40	43	0.88	46	0.45
3	1228	0.37	778	0.35	58	0.95	67	0.35
4	242	0.34	150	0.32	13	0.38	8	0.30
5	88	0.41	32	0.25	5	0.98	2	0.36
6 (most rural)	24	0.29	15	0.28	3	1.61	2	0.90
**Perinatal factors**								
*Prenatal factors*								
Maternal pre-pregnancy body mass index								
< 18.5	330	0.44	289	0.45	20	0.44	22	0.47
18.5–24.9	1986	0.34	2493	0.34	110	0.37	177	0.37
25.0–29.9	1316	0.35	1090	0.35	56	0.46	87	0.37
≥ 30	1331	0.41	905	0.39	40	0.54	80	0.42
Prenatal care^c^								
Inadequate	1443	0.39	556	0.35	122	0.62	82	0.47
Intermediate	265	0.20	232	0.15	14	0.22	19	0.23
Adequate/adequate plus	3495	0.38	4238	0.39	117	0.33	274	0.38
Smoked during pregnancy								
Yes	657	0.68	162	0.49	49	1.52	44	0.83
No	4884	0.36	5042	0.36	217	0.35	362	0.38
*Pregnancy factors*								
Parity and mode of birth								
Primiparous vaginal	457	0.13	462	0.11	20	0.10	23	0.11
Multiparous vaginal	783	0.13	540	0.10	41	0.17	43	0.11
Primiparous primary cesarean	1022	0.68	1592	0.74	57	0.69	102	0.84
Multiparous primary cesarean	1580	1.75	1597	1.88	69	2.00	132	2.08
Repeat cesarean	1696	0.73	1072	0.56	77	0.84	100	0.66
Pregnancy type								
Singleton	4285	0.30	3156	0.23	217	0.35	269	0.28
Multiple gestation	1256	3.92	2048	3.51	49	2.85	137	4.40
*Obstetric co-morbidities*								
Hypertension								
No HTN	3186	0.24	2396	0.18	161	0.26	231	0.25
Preexisting HTN without preeclampsia	214	1.59	159	0.87	10	3.00	16	1.32
Preexisting HTN with preeclampsia	416	5.95	320	4.26	17	8.72	27	4.83
Gestational HTN without preeclampsia	166	0.53	150	0.46	8	0.73	13	0.54
Gestational HTN with preeclampsia	1226	2.58	1422	3.09	51	3.17	89	3.26
Diabetes								
No diabetes	4531	0.34	4198	0.03	237	0.39	359	0.39
Preexisting diabetes	270	1.96	172	1.66	8	3.08	7	0.84
Gestational diabetes	740	0.62	834	0.63	21	0.62	40	0.59

Dual burden defined as concomitant severe maternal morbidity or maternal in-hospital death and preterm birth (< 37 weeks). Severe maternal morbidity was determined according to the CDC algorithm + birth certificate: 1) in-hospital deaths, regardless of length of stay; 2) #19–25 without regard to length of stay; 3) #1–18 and length of stay restriction, ≥ 90th percentile for type of birth with length of stay calculated separately for vaginal birth, primary cesarean, and repeat cesarean; and #1–25 on postpartum readmission regardless of length of stay)

*BMI* body mass index, *HTN* hyptertension

^a^Other insurance includes: Indian Health Service, CHAMPUS or TRICARE, other local, state, or federal insurance, or charity

^b^Federal Information Processing Standard categories

^c^Kotelchuck Adequacy of Prenatal Care Utilization Index (Harrison & Goldenberg, 2016)

**Table 4:** Gestational age and preterm indication among births with dual burden of severe maternal morbidity and preterm birth by insurance type, California 2007–2012

Characteristic	Medi-cal		Private		Uninsured		Other^a^	
	n = 1,462,463		n = 1,431,956		n = 64,507		n = 100,149	
	n	%	n	%	n	%	n	%
< 28 weeks gestational age	7,742	0.53	6,670	0.47	552	0.86	597	0.60
PPROM	2,566	0.18	2,344	0.16	176	0.27	164	0.16
Preterm labor without PPROM	4,557	0.31	3,703	0.26	316	0.49	391	0.39
Medically indicated	457	0.03	522	0.04	43	0.07	31	0.03
Unknown	162	0.01	101	0.01	17	0.03	11	0.01
28 to < 32 weeks gestational age	11,240	0.77	11,411	0.80	617	0.96	819	0.82
PPROM	3,039	0.21	3,411	0.24	171	0.27	211	0.21
Preterm labor	6,506	0.44	6,389	0.45	335	0.52	515	0.51
Medically indicated	1,362	0.09	1,432	0.10	86	0.13	80	0.08
Unknown	333	0.02	179	0.01	25	0.04	13	0.01
32 to < 37 weeks gestational age	106,640	7.29	108,643	7.59	4,972	7.71	7,674	7.66
PPROM	19,358	1.32	25,344	1.77	985	1.53	1,295	1.29
Preterm labor	58,227	3.98	56,327	3.93	2,576	3.99	4,468	4.46
Medically indicated	20,578	1.41	21,509	1.50	858	1.33	1,400	1.40
Unknown	8,477	0.58	5,463	0.38	553	0.86	511	0.51
37 to 38 weeks gestational age	408,302	27.90	377,844	26.39	18,506	28.69	28,362	28.32
39 to 42 weeks gestational age	927,227	63.40	926,575	64.71	39,795	61.69	62,610	62.52
43 to 44 weeks gestational age	1,312	0.09	812	0.06	65	0.10	87	0.09

Dual burden defined as concomitant severe maternal morbidity or maternal in-hospital death and preterm birth (< 37 weeks). Severe maternal morbidity was determined according to the CDC algorithm + birth certificate: (1) in-hospital deaths, regardless of length of stay; (2) #19–25 without regard to length of stay; (3) #1–18 with length of stay restriction (≥ 90th percentile for type of birth with length of stay calculated separately for vaginal birth, primary cesarean, and repeat cesarean); or (4)#1–25 on postpartum readmission regardless of length of stay). Gestational age and prematurity categories are provided for descriptive purposes only and are not included in subsequent tables or as covariates in adjusted analyses*.* PPROM: Preterm premature rupture of the membranes

^a^Other insurance includes: Indian Health Service, CHAMPUS or TRICARE, other local, state, or federal insurance, or charity

### Dual Burden of Severe Maternal Morbidity and Preterm Birth by Maternal Characteristics across Insurance Type

Women with preexisting and obstetric comorbidities had the highest rates of dual burden across insurance types, including women with preexisting hypertension with preeclampsia (range 4.3–8.7%), gestational hypertension with preeclampsia (range 2.6–3.3%), preexisting hypertension without preeclampsia (range 0.9–3.0%), and preexisting diabetes (range 0.8–3.1%). Similarly high rates of dual burden were identified in women with multiple gestation and cesarean birth.

Maternal characteristics exhibiting the largest differences in the rate of dual burden of SMM and preterm birth rate across insurance type included preexisting and obstetric comorbidities, multiple gestations, smoking during pregnancy, other race/ethnicity, and urban/rural status. The highest rates of dual burden were generally observed among uninsured women. For example, women with preexisting hypertension with and without preeclampsia, dual burden rates were lowest for women with Medi-Cal (6.0% and 1.6%, respectively) and highest for uninsured women (8.7% and 3.0%, respectively). Among the characteristics with relatively large differentials across payor type, multiple gestation was the only characteristic in which uninsured women had the lowest rates, at 2.9%, with the highest rate at 4.4% among other-insured women (Table 2).

### Relative Risk of Dual Burden of Severe Maternal Morbidity and Preterm Birth by Maternal Characteristics Across Insurance Type

Obstetric comorbidities, pregnancy type, parity, and mode of birth conferred the largest adjusted relative risks of dual burden across all delivery payor types and showed variation in effect magnitude across payor existed (Table 5). Elevated risks associated with preexisting hypertension, both with and without preeclampsia, were lowest among privately insured women and highest among uninsured women. For example, preexisting hypertension without preeclampsia was associated with a 2.7-fold (aRR 2.65, 95% CI 2.21–3.19) increased dual burden risk among privately insured women to a 7.3-fold (aRR 7.27, 95% CI 3.64–14.51) risk increase among uninsured women. The range for preexisting hypertension with preeclampsia ranged from ninefold to 16-fold among private and uninsured women, respectively. Increased risk of dual burden associated with gestational hypertension without preeclampsia had a smaller effect and range across insurance payor, from aRR 1.45 (95% CI 1.15–1.82) among privately insured women to 1.89 (95% CI 0.86–4.24) among uninsured women. Increased risk of dual burden associated with gestational hypertension with preeclampsia ranged from 6.9-fold among privately insured women (aRR 6.93, 95% CI 6.28–7.65) to 8.1-fold (aRR 8.13, 95% CI 5.35–12.35) among uninsured women; no significant differences in effect size by insurance type were identified.

**Table 5:** Adjusted relative risk of dual burden of severe maternal morbidity and preterm birth by sociodemographic, pregnancy, and obstetric factors, stratified by insurance Payor, California 2007–2012

Characteristic	Medical	Private	Uninsured	Other^a^
	n = 1,462,463	n = 1,431,956	n = 64,507	n = 100,149
	aRR (95% CI)	aRR (95% CI)	aRR (95% CI)	aRR (95% CI)
**Sociodemographic characteristics**				
Maternal age				
< 18	1.12 (0.95–1.33)	1.63 (1.16–2.29)	1.50 (0.78–2.87)	1.75 (1.01–3.01)
18–25	Reference	Reference	Reference	Reference
26–35^b^	1.11 (1.03–1.19)	0.99 (0.91–1.08)	0.62 (0.43–0.89)	1.23 (0.90–1.69)
> 35^c,e,g^	1.45 (1.31–1.60)	1.30 (1.17–1.46)	0.86 (0.60–1.25)	1.62 (1.10–2.37)
Race/ethnicity				
White non-Hispanic	Reference	Reference	Reference	Reference
Hispanic ^b,c,e,g^	1.03 (0.93–1.14)	1.24 (1.13–1.37)	0.72 (0.52–1.01)	1.24 (0.92–1.68)
Black, non-Hispanic	1.91 (1.66–2.18)	1.93 (1.66–2.25)	1.55 (1.00–2.44)	2.16 (1.55–3.01)
Asian, non-Hispanic^c,d,e^	1.09 (0.89–1.33)	1.26 (1.11–1.43)	0.46 (0.20–1.05)	0.83 (0.53–1.33)
Other, non-Hispanic^c^	1.28 (1.12–1.46)	1.47 (1.29–1.67)	1.08 (0.68–1.71)	1.11 (0.67–1.84)
Education				
< 12 years^b,d^	0.95 (0.87–1.03)	1.22 (1.06–1.41)	1.12 (0.85–1.49)	1.05 (0.75–1.46)
12 years	Reference	Reference	Reference	Reference
> 12 years	0.96 (0.88–1.05)	0.85 (0.77 -0.93)	0.69 (0.45–1.04)	0.80 (0.60–1.05)
Urban/rural status^h^				
1 (most urban)	Reference	Reference	Reference	Reference
2^c,d^	1.10 (0.89–1.35)	1.13 (1.00–1.29)	1.91 (1.16–3.15)	1.15 (0.64–2.04)
3^c,d^	1.01 (0.84–1.20)	1.01 (0.84–1.21)	1.86 (1.11–3.10)	1.05 (0.72–1.54)
4	0.89 (0.71–1.11)	0.92 (0.68–1.24)	0.89 (0.42–1.92)	0.79 (0.32–1.90)
5	1.07 (0.66–1.74)	0.79 (0.46–1.37)	1.91 (0.59–6.14)	^h^
6 (most rural)	0.72 (0.45–1.16)	0.82 (0.38–1.76)	^h^	^h^
**Prenatal factors**				
Maternal pre-pregnancy BMI				
< 18.5	1.33 (1.17–1.52)	1.44 (1.23–1.69)	1.22 (0.77–1.93)	1.49 (0.96–2.31)
18.5–24.9	Reference	Reference	Reference	Reference
25.0–29.9	0.81 (0.75–0.87)	0.87 (0.79–0.96)	0.96 (0.69–1.34)	0.78 (0.60–1.02)
≥ 30	0.68 (0.63–0.74)	0.77 (0.69–0.87)	0.81 (0.54–1.20)	0.72 (0.53–0.98)
Prenatal care^i^				
Inadequate^c,e^	1.12 (1.02–1.21)	1.05 (0.93–1.19)	1.58 (1.14–2.21)	1.37 (0.99–1.89)
Intermediate	0.74 (0.60–0.91)	0.66 (0.55–0.78)	0.65 (0.29–1.48)	0.95 (0.59–1.55)
Adequate/adequate plus	Reference	Reference	Reference	Reference
Smoked during pregnancy^c^^,e,g^	1.58 (1.40–1.80)	1.20 (0.91–1.57)	2.93 (1.96–4.37)	1.51 (1.03–2.21)
**Pregnancy factors**				
Parity and mode of birth				
Primiparous vaginal	Reference	Reference	Reference	Reference
Multiparous vaginal^b,c,e^	0.90 (0.80–1.01)	0.76 (0.68–0.86)	1.70 (1.00–2.88)^g^	1.03 (0.64–1.64)
Primiparous primary cesarean^f^	3.07 (2.72–3.47)	3.41 (3.01–3.87)	4.29 (2.31–7.97)	5.38 (3.53–8.21)
Multiparous primary cesarean^c,e,f^	8.21 (7.05–9.57)	7.03 (5.95–8.31)	19.35 (10.33–36.33)	12.77 (8.24–19.78)
Repeat cesarean^c,e^	3.92 (3.47–4.42)	3.33 (2.91–3.80)	6.89 (4.22–11.26)	4.43 (2.88–6.80)
Pregnancy type				
Singleton	Reference	Reference	Reference	Reference
Multiple gestation^c,e,g^	6.00 (5.28–6.82)	6.93 (6.24–7.70)	3.80 (2.54–5.67)	7.94 (5.99–10.52)
**Obstetric co-morbidities**				
Hypertension				
No HTN	Reference	Reference	Reference	Reference
Preexisting HTN without preeclampsia^b,e^	3.84 (3.28–4.51)	2.65 (2.21–3.19)	7.27 (3.64–14.51)	3.26 (1.92–5.53)
Preexisting HTN with preeclampsia^b^	12.98 (11.21–15.03)	9.12 (7.62–10.92)	15.87 (9.14–27.57)	9.13 (5.76–14.46)
Gestational HTN without preeclampsia	1.86 (1.56–2.22)	1.45 (1.16–1.82)	1.89 (0.84–4.24)	1.57 (0.79–3.12)
Gestational HTN with preeclampsia	7.60 (6.79–8.50)	6.93 (6.28–7.65)	8.13 (5.35–12.35)	8.02 (5.64–11.42)
Diabetes				
No diabetes	Reference	Reference	Reference	Reference
Gestational diabetes^d^	2.54 (2.16–2.99)	2.62 (2.21–3.11)	3.43 (1.76–6.68)	1.30 (0.50–3.38)
Preexisting diabetes^d^	1.14 (1.05–1.24)	1.16 (1.06–1.27)	1.20 (0.74–1.97)	1.00 (0.72–1.39)

*BMI* body mass index, *HTN* hypertension, *aRR* adjusted relative risks; Relative risks adjusted by all other variables in the table

^a^Other insurance includes: Indian Health Service, CHAMPUS or TRICARE, other local, state, or federal insurance, or charity

^b^pairwise comparison Medi-Cal vs. private p < 0.05

^c^pairwise comparison Medi-Cal vs. uninsured p < 0.05

^d^pairwise comparison Medi-Cal vs. other p < 0.05

^e^pairwise comparison private vs. uninsured p < 0.05

^f^pairwise comparison private vs. other p < 0.05

^g^pairwise comparison uninsured vs. other p < 0.05

^h^Federal Information Processing Standard categories

^i^Kotelchuck Adequacy of Prenatal Care Utilization Index (Harrison & Goldenberg, 2016)

Multiple gestation was associated with increased risk of dual burden across all insurance payors; however, the effect associated with multiple gestation was significantly lower among uninsured women (aRR 3.80, 95% CI 2.54–5.67) than among women covered by Medi-Cal, private, and other insurance (aRR range 6.0–7.94).

Cesarean births were associated with significantly higher risk of dual burden, particularly multiparous primary cesarean delivery which ranged from sevenfold (aRR 7.03, 95% CI 5.95–8.31) among privately insured women to 19-fold (aRR 19.35, 95% CI 10.33–36.33) among uninsured women. Dual burden associated with repeat cesarean was also significantly higher among uninsured women, at 6.89 (95% CI 4.22–11.26) compared to primiparous women with vaginal births.

Risk differences according to insurance type, smoking during pregnancy, and maternal race/ethnicity were present but attenuated. Risk of dual burden for Black women compared to white non-Hispanic women was significantly elevated among women with Medi-Cal, private insurance, and other insurance. However, no statistically significant increased risk was identified among uninsured women.

## Discussion

Risk of dual burden of SMM and preterm birth was highest among women with no health insurance or other health insurance. Within each insurance type, the primary independent predictors of dual burden were generally consistent with previous studies (Lyndon et al., 2019). However, in this first known study to explore the magnitude of effect of sociodemographic and perinatal factors across insurance types, we identified important differences in magnitude of dual burden risk. Other literature investigating disparities in maternal morbidity by insurance status has been largely limited to comparisons between Medicaid versus privately insured women. The results of the study support the need to focus on the broader range of insurance types (Brandon et al., 2009; Greiner et al., 2018; Fingar et al., 2018; Lipkind et al., 2009).

Risk factors consistently influencing dual burden risk across insurance type of obstetric comorbidities, parity, and mode of birth were generally the highest among uninsured women, followed by women with Medi-Cal. However, the statistical significance of these differences was inconsistent. This pattern was different for multiple gestation where risks were significantly lower for uninsured women compared to all other insurance categories. This finding is puzzling and warrants further inquiry into profiles of multiple gestation patients across insurance categories. This finding may be partially explained by the links between multiple gestation, insurance type, and assisted reproductive technology. In California, 16% of multiple gestation births are through assisted reproductive technology (Sunderam et al., 2017), and access to assisted reproductive technology differs by insurance coverage (Kulkarni et al., 2013; Provost et al., 2016). Similarly, uninsured women were also the only payor group without an advanced maternal age-related increase in dual burden (Lean et al., 2017).

Substantial disparities in dual burden by race and ethnicity have been identified elsewhere, with significantly higher risk among racially and ethnically minoritized groups compared to non-Hispanic white women, including a two-fold increased risk for non-Hispanic Black women (Lyndon et al., 2019). Our current findings are consistent with the literature linking structural racism to adverse perinatal outcomes (Burris & Parker, 2021; Chambers et al., 2018; Lyndon et al., 2019; Shrimali et al., 2020; Taylor, 2020), with significantly increased risk of dual burden for Black compared to non-Hispanic white women for women with all insurance types except women with no insurance. The absence of a statistically significant Black-white disparity among uninsured women may reflect the greater level of disadvantage among this population overall.

Interpreting these findings for clinical intervention is not straightforward; instead, the findings suggest areas for additional research to further disentangle these results. Insurance status likely represents a variety of characteristics or exposures that may themselves be linked to risk of dual burden. For example, insurance status may be used as a rough proxy measure for socioeconomic status and is patterned by other characteristics such as race/ethnicity and health status (Artiga et al., 2019; Barnett et al., 2017; Sohn 2017), which are patterned by racism and socioeconomic discrimination that increase risk of adverse perinatal outcomes (Chambers et al., 2018, 2019; Shrimali et al., 2020). Furthermore, continuity of insurance coverage may be unstable during the perinatal period, with interruptions in coverage resulting from changing eligibility status (Daw & Sommers 2018). Severe pre-existing or prenatal morbidities may influence an insurance payor at birth through structuring a woman’s eligibility for public insurance (Martin & Bellux, 2019). These observations suggest that baseline pre-pregnancy and pregnancy health status may differ in important ways across insurance payor; although we adjusted for a variety of conditions and potential contributors to pre-pregnancy and pregnancy health, our analysis remains limited by the administrative data available.

While we have no direct comparison for the findings of this analysis, research in other areas that have examined health outcomes by payor status generally support improved health outcomes for individuals covered by private insurance, with individuals with public insurance considered to reflect greater disease severity. For example, a retrospective analysis of preventive care in a non-Medicaid expansion state found Medicaid-insured or uninsured women had fewer well-woman visits prior to giving birth and higher emergency department visits during pregnancy, whereas privately insured women were more likely to initiate prenatal care in the first trimester, receive adequate-plus- prenatal care, and have a postpartum checkup within six weeks of delivery (Taylor et al., 2002). Similarly, among older adults undergoing cancer-related surgery, Medicaid covered care was associated with longer lengths of stay and higher inpatient costs for colorectal, non-small cell lung cancer, and breast cancer (Bradley et al., 2012). Some have postulated that different inpatient experiences and outcomes across insurance payor may be due to differences in the clinical care provided. For example, the Kaiser Family Foundation hypothesizes that hospitals may compensate for rising costs by performing certain discretionary services for financially lucrative patients while economizing on services provided to low-income patients, for example, discretionary procedures such as breast construction; however, this literature is limited (Bradley et al., 2012). It is likely that the differences observed in our study and others reflect some combination of these two explanations. Further research on variation in quality of maternity care across insurance type would help us better understand the differences found in risk factors across insurance type.

The current analysis is limited by the administrative nature of the dataset used. For example, the potential for differential misclassification of morbidity predictors or status exists (Dietz et al., 2003; Kane & Sappenfield, 2014; Mallinson & Ehrenthal, 2019), and a variety of potentially important behavioral risk factors or care processes were unable to be precisely measured or included. Any misclassification in morbidity status is likely to result in a conservative analysis through underreporting (Main et al., 2016), particularly due to our use of a 90th percentile length of stay restriction,(Callaghan et al., 2012) and SMM algorithm validated for population-level measurement (Lydon-Rochelle et al., 2005; Yasmeen et al., 2006). Our findings are based on population-level data from a single large state accounting for > 12% of all U.S. births. They may not be generalizable to areas with differing demographics, risk profiles, and health care systems. Due to the limited descriptors of the other insurance category, the results of this group may be harder to interpret.

The observed differences in the magnitude of the effects of sociodemographic, pregnancy-related, and obstetric factors by insurance type raise concerns that important differences in care quality exist by insurance payor, which may increase women’s risks of the dual burden of SMM and preterm birth. Our findings are consistent with previous studies demonstrating differences in care quality by insurance type for other conditions and suggest that attention to ensuring that all birthing people and infants receive high-quality peripartum care regardless of insurance payor is needed to improve maternal and neonatal health.

## Supplementary Information

Below is the link to the electronic supplementary material.

Supplementary file1 (DOCX 47 kb)

## Footnotes

1 Other insurance includes: Indian Health Service, CHAMPUS or TRICARE, other local, state, or federal insurance, or charity.
