# Supplementary material for: Risk Factors for Dual Burden of Severe Maternal Morbidity and Preterm Birth by Insurance Type in California
Source: Matern Child Health J. 2022 Jan 18;26(3):601–13. doi: 10.1007/s10995-021-03313-1 (PMC8917014; doi:10.1007/s10995-021-03313-1)
Supplement: Supplementary file 1 — Supplementary file1 (DOCX 47 kb) [file 10995_2021_3313_MOESM1_ESM.docx]

SUPPLEMENTAL FILES

Table S1. Centers for Disease Control Severe Maternal Morbidity Indicators and Corresponding ICD-9-CM Codes^34^.

| Severe Maternal Morbidity Indicator | ICD-9-CM Codes | Diagnosis Code | Procedure Code |
| --- | --- | --- | --- |
| 1. Acute myocardial infarction^a^ | 410.xx | x |  |
| 2. Acute renal failure | 584.x, 669.3x | x |  |
| 3. Adult respiratory distress syndrome | 518.5, 518.81, 518.82, 518.84,799.1 | x |  |
| 4. Amniotic fluid embolism | 673.1x | x |  |
| 5. Aneurysm | 441.xx | x |  |
| 6. Cardiac arrest/ventricular fibrillation^a^ | 427.41, 427.42, 427.5 | x |  |
| 7. Disseminated intravascular coagulation | 286.6, 286.9, 666.3x | x |  |
| 8. Eclampsia | 642.6x | x |  |
| 9. Heart failure during procedure or surgery^a^ | 669.4x, 997.1 | x |  |
| 10. Internal injuries of thorax, abdomen, and pelvis | 860.xx—869.xx | x |  |
| 11. Intracranial injuries | 800.xx, 801.xx, 803.xx, 804.xx, 851.xx-854.xx | x |  |
| 12. Puerperal cerebrovascular disorders | 430, 431, 432.x, 433.xx, 434.xx, 436, 437.x, 671.5x, 674.0x, 997.2, 999.2 | x |  |
| 13. Pulmonary edema | 428.1, 518.4 | x |  |
| 14. Severe anesthesia complications | 668.0x, 668.1x, 668.2x | x |  |
| 15. Sepsis | 038.xx, 995.91, 995.92 | x |  |
| 16. Shock | 669.1x, 785.5x, 995.0, 995.4, 998.0 | x |  |
| 17. Sickle cell anemia with crisis | 282.62, 282.64, 282.69 | x |  |
| 18. Thrombotic embolism | 415.1x, 673.0x, 673.2x, 673.3x, 673.8x | x |  |
| 19. Blood transfusion | 99.0x |  | x |
| 20. Cardio monitoring^a^ | 89.6x |  | x |
| 21. Conversion of cardiac rhythm^a^ | 99.6x |  | x |
| 22. Hysterectomy | 68.3x-68.9 |  | x |
| 23. Operations on heart and pericardium^a^ | 35.xx, 36.xx, 37.xx, 39.xx |  | x |
| 24. Temporary tracheostomy | 31.1 |  | x |
| 25. Ventilation | 93.90, 96.01-96.05, 96.7x |  | x |

*Source:* <http://www.cdc.gov/reproductivehealth/maternalinfanthealth/severematernalmorbidity.html> *Accessed* July 14, 2016. *Note:* ICD-9-CM, International Classification of Diseases, Ninth Revision, Clinical Modification. ^a^Indicators comprising ‘combined cardiac morbidity’.
